# UEG and EAES rapid guideline: Systematic review, meta-analysis, GRADE assessment and evidence-informed European recommendations on TaTME for rectal cancer

**DOI:** 10.1007/s00464-022-09090-4

**Published:** 2022-02-25

**Authors:** Marco Milone, Michel Adamina, Alberto Arezzo, Nona Bejinariu, Luigi Boni, Nicole Bouvy, F Borja de Lacy, Raphaëla Dresen, Konstantinos Ferentinos, Nader K. Francis, Joe Mahaffey, Marta Penna, George Theodoropoulos, Katerina Maria Kontouli, Dimitris Mavridis, Per Olav Vandvik, Stavros A. Antoniou

**Affiliations:** 1 Department of Clinical Medicine and Surgery, University “Federico II” of Naples, Naples, Italy; 2 Department of Surgery, Clinic of Visceral and Thoracic Surgery, Cantonal Hospital Winterthur, Winterthur, Switzerland; 3 Department of Biomedical Engineering, Faculty of Medicine, University of Basel, Allschwil, Switzerland; 4 Department of Surgical Sciences, University of Torino, Torino, Italy; 5 Department of Pathology, Santomar Oncodiagnostic, Cluj-Napoca, Romania; 6 Department of Surgery, Fondazione IRCCS – Ca’ Granda - Ospedale Maggiore Policlinico University of Milan, Milan, Italy; 7 Department of Surgery, Maastricht University Medical Center, Maastricht, Netherlands; 8 Department of Gastrointestinal Surgery, Institute of Digestive and Metabolic Diseases, Hospital Clinic of Barcelona, Barcelona, Spain; 9 University Hospitals Leuven, Leuven, Belgium; 10 Department of Radiation Oncology, German Oncology Center, Limassol, Cyprus; 11 Present Address: European University Cyprus, Nicosia, Cyprus; 12 Department of General Surgery, Yeovil District Hospital NHS Foundation Trust, Yeovil, UK; 13 Fight Colorectal Cancer, Springfield, MO USA; 14 Oxford University Hospitals, Oxford, UK; 15 First Department of Propaedeutic Surgery of Athens, Medical School, National and Kapodistrian University of Athens, Athens, Greece; 16 Department of Primary Education, School of Education, University of Ioannina, Ioannina, Greece; 17 Faculté de Médecine, Université Paris Descartes, Paris, France; 18 Department of Health Management and Health Economics, University of Oslo, Oslo, Norway; 19 Surgical Department, Mediterranean Hospital of Cyprus, Limassol, Cyprus

**Keywords:** Rectal cancer, TaTME, Transanal TME, Clinical practice guideline, GRADE, EAES

## Abstract

**Background:**

Evidence and practice recommendations on the use of transanal total mesorectal excision (TaTME) for rectal cancer are conflicting.

**Objective:**

We aimed to summarize best evidence and develop a rapid guideline using transparent, trustworthy, and standardized methodology.

**Methods:**

We developed a rapid guideline in accordance with GRADE, G-I-N, and AGREE II standards. The steering group consisted of general surgeons, members of the EAES Research Committee/Guidelines Subcommittee with expertise and experience in guideline development, advanced medical statistics and evidence synthesis, biostatisticians, and a guideline methodologist. The guideline panel consisted of four general surgeons practicing colorectal surgery, a radiologist with expertise in rectal cancer, a radiation oncologist, a pathologist, and a patient representative. We conducted a systematic review and the results of evidence synthesis by means of meta-analyses were summarized in evidence tables. Recommendations were authored and published through an online authoring and publication platform (MAGICapp), with the guideline panel making use of an evidence-to-decision framework and a Delphi process to arrive at consensus.

**Results:**

This rapid guideline provides a weak recommendation for the use of TaTME over laparoscopic or robotic TME for low rectal cancer when expertise is available. Furthermore, it details evidence gaps to be addressed by future research and discusses policy considerations. The guideline, with recommendations, evidence summaries, and decision aids in user-friendly formats can also be accessed in MAGICapp: https://app.magicapp.org/#/guideline/4494.

**Conclusions:**

This rapid guideline provides evidence-informed trustworthy recommendations on the use of TaTME for rectal cancer.

**Supplementary Information:**

The online version contains supplementary material available at 10.1007/s00464-022-09090-4.

Colorectal cancer affects a substantial proportion of the general population, with a lifetime risk of 4.3% for men and 4% for women [1]. Rectal cancer accounts for 23–32% of colorectal malignancies [2]. The anatomy of the rectum makes surgical treatment of low rectal cancer challenging. Laparoscopic surgery has been found to likely result in similar 5-year oncological outcomes and reduced minor morbidity compared to open surgery [3], whereas it facilitates improved visualization for dissection deep in the pelvis. Robotic surgery has been suggested to confer further technical advantages [4].

Rectal dissection is, however, challenging in low-lying tumors and in patients with unfavorable anatomy, such as male and obese individuals. Transanal total mesorectal excision (TaTME) has been developed as an alternative technique, that allows down-to-up dissection of the rectum and perineal dissection of the mesorectum without the need for deep abdominal dissection. It has been hypothesized that this approach may improve the quality of the specimen [5].

TaTME has attracted much attention over the past few years and it has stimulated a debate around its safety and efficacy [6–10]. Moreover, consensus panels and practice statements have provided conflicting recommendations [11–14]. Under consideration of dissenting views and opinions, and taking into account EAES members' preferences who have prioritized colorectal cancer as a guideline topic in an online survey [15], UEG and EAES have sponsored the development of this project.

## Objective

The objective of this rapid guideline was to develop reliable, trustworthy, pertinent, evidence-informed recommendations based on state-of-the-art guideline development methodology on the use of TaTME versus laparoscopic or robotic surgery in patients with rectal cancer.

## Methods

The protocol of this rapid guideline is available online [16]. It was reported in accordance with AGREE II and it was developed following GRADE, Institute of Medicine and Guidelines International Network standards [17–19]. Furthermore, we adhered to GRADE guidance published in the Journal of Clinical Epidemiology as part of a series of articles detailing and updating the GRADE methodology. This guideline was facilitated with the online authoring and publication platform MAGICapp.

This is an outline of the methodology; more detailed information is provided in MAGICapp (https://app.magicapp.org/#/guideline/4767) and in the Appendix; complete datasets are available online [20].

### Steering group

The guideline steering group consisted of a general surgeon performing laparoscopic, robotic and transanal TME (coordinator, MM), a certified guideline methodologist with vast experience in evidence outreach, synthesis, assessment and guideline development, (supervisor, SAA); biostatisticians (KMK, DM); and a GRADE external auditor (POV). All members of the steering group disclosed no conflicts, direct or indirect [20].

### Guideline panel

The guideline panel consisted of four general surgeons, a radiation oncologist, a radiologist, a pathologist, and a patient advocate (AA, NB, NB, ED, KF, NKF, JM, GT). The patient advocate resides in the USA and was nominated by the European Patients' Forum, a non-profit umbrella organization of patient organizations across Europe. Panel members watched a short video tutorial outlining the guideline development methodology. The composition of panel members aimed to be representative of different parts of Europe, both genders, different age groups, and academic/non-academic surgical practice. Panel members disclosed no direct nor indirect conflicts [20]. External advisors were surgeons with clinical experience and/or research focus on TaTME (MA, LB, FBdL, MP). They were consulted throughout the guideline development process, but they did not vote on the direction, the strength and the wording of the recommendations.

### Guideline questions

1. Should TaTME versus laparoscopic TME be preferred for the treatment of rectal cancer?
2. Should TaTME versus robotic TME be preferred for the treatment of rectal cancer?

### Protocol

A protocol was developed a priori by the steering group [16]. The protocol draft was made publicly available through the EAES website and EAES members were invited through various channels to comment on the content. The guideline questions and outcomes were refined in collaboration with the guideline panel members, whereas EAES members' comments were considered and several were addressed (see Appendix). Amendments to the protocol with justifications are provided in the Appendix.

### Rating the importance of outcomes

The importance of outcomes was rated by the panel members using the GRADE scale [21]. The classification of outcomes into each of the three categories (not important, important, critical) was made by the steering group under consideration of panel members' ratings available online [20].

We considered the importance of outcomes as follows:

1. 30-day or in-hospital mortality: critical
2. 30-day complications Clavien-Dindo ≥ 3 (major morbidity): critical
3. 30-day complications Clavien-Dindo ≤ 2 (minor morbidity): important
4. Anastomotic leakage: critical
5. Completeness of TME: critical
6. Disease recurrence at 2 years: critical
7. 5-year overall survival: critical
8. 5-year disease-free survival: critical
9. Low anterior resection syndrome: critical
10. Quality of life: critical

### Setting minimal important differences

The evidence-to-decision framework was set within a fully contextualized approach [7]. An anonymous web-based survey of panel members was performed to define minimal important differences. The results of the survey are available online [20].

Under consideration of panel's responses, the following minimal important differences were considered:

1. 30-day or in-hospital mortality: 10 per 1000
2. 30-day complications Clavien-Dindo ≥ 3 (major morbidity): 10–50 per 1000
3. 30-day complications Clavien-Dindo ≤ 2 (minor morbidity): 50–100 per 1000
4. Anastomotic leakage: 25 per 1000
5. Completeness of TME: 25–50 per 1000
6. Disease recurrence at 2 years: 25–50 per 1000
7. 5-year overall survival: 10–50 per 1000
8. 5-year disease-free survival: 10–25 per 1000
9. Low anterior resection syndrome: 50 per 1000
10. Quality of life: score 5–10 out of 100

### Search strategy

One strategy was developed for both guideline questions because of their affinity. The databases of Medline, EMBASE and OpenGrey were searched. The search syntaxes are available online [20].

### Study selection

Titles and/or abstracts were screened (first level) and full text articles were scrutinized (second level) to identify eligible studies in duplicate (MM, SAA). Inclusion criteria were adult patients with adenocarcinoma of the rectum, TaTME compared with laparoscopic/robotic TME. Exclusion criteria were single incision and open surgery.

### Risk of bias assessment

RoB-2 and ROBINS-I were used for risk of bias assessment in RCTs and cohort studies with a comparative arm, respectively [22, 23]. Relevant considerations are provided in the Appendix.

### Statistical analysis

We conducted random effects meta-analyses to quantitatively synthesize the evidence for the guideline questions since we expected much variation in the PICO criteria across studies [25]. We explored heterogeneity via the *I*^*2*^ statistic that describes the percentage of the variability of effect estimates that is due to heterogeneity rather than sampling error. We further explored heterogeneity by computing the *Q*-statistic and the 95% predictive intervals that show the plausible range of effect size values for a future trial. All the analyses were performed in *R* statistical package version 4.0.3 using the *meta* package. All statistical analyses were performed independently by the statisticians' group with no involvement of the steering group or panel members.

### Evidence tables

We constructed GRADE evidence profiles of certainty for each outcome separately using MAGICapp. The certainty of evidence is determined by the risk of bias across studies, incoherence, indirectness, imprecision, publication bias and other parameters [26]. We used the most recent GRADE methodology to decide on the certainty of the body of evidence from RCTs and observational studies using RoB-2 and ROBINS-I, which recommends using the judgment of high certainty of evidence at baseline and downgrading due to risk of bias of RCTs and observational studies [27]. Minimal important differences determined in advance through a survey of panel members were used to inform judgements about precision and coherence. When very low certainty evidence on an outcome was found, we used a ‘systematic observation form to retrieve expert-based evidence’ as previously described [28]. Evidence tables for Q1 were informed by the systematic observation form (relevant data are available online [20]), whereas experience with robotic TME was limited to provide substantial expert-based observation evidence.

### Evidence-to-decision framework

The panel discussed the evidence within a GRADE evidence-to-decision framework coordinated by the guideline methodologist using MAGICapp. A formal anonymous Delphi process was carried out to finalize the judgements. A total of two online meetings were required.

### Developing recommendations

Based on the evidence-to-decision framework, the panel anonymously voted on the strength and the direction of the recommendations through MAGICapp. There was unanimous consensus on the strength and the direction of the recommendations, whereas minor dissenting opinions on the wording were noted and reported accordingly in this manuscript.

## Results

Some 822 records and 46 full text articles were screened, out of which 17 met the eligibility criteria. Sixeen studies addressed Q1 [29–44] and one study addressed Q2 [45]. The study selection flowchart and considerations on record selection, and risk of bias summaries are provided in the Appendix; detailed files including discarded records with reasons, and risk of bias judgements with detailed justifications are available online [20]. Forest plots of meta-analyses are provided on MAGICapp.

Data on disease-free and overall survival were provided by one study only; local recurrence at 2 years was provided by two studies [40, 44]; however, the study was at critical risk of bias with regard to this outcome and did therefore not enter the analysis as per ROBINS-I methodology [23]. Low anterior resection syndrome and quality of life were reported by only a few studies [35, 43].

Several articles addressed parameters pertinent to the evidence-to-decision framework [46–53].

### Recommendation – TaTME versus laparoscopic TME

**We suggest TaTME over laparoscopic TME if expertise is available.** *Weak recommendation*

*Rationale*

The panel identified some evidence of benefit in critical outcomes with TaTME and no evidence of harm; nevertheless, the overall certainty of the evidence was very low, primarily due to confounding bias and imprecision of effect estimates, whereas evidence on some critical outcomes, primarily survival outcomes, was very low. Substantial variability in patient values and preferences is anticipated and patient aids might be useful in this context. There is uncertainty around the use of resources, whereas equity might be reduced, due to lack of widespread expertise and longer use of operating room resources, at least during the early stages of implementation. The panel considered the intervention to be acceptable to key stakeholders, whereas feasibility was considered to vary and depend on annual volume of cases and centralization of care. An important parameter which determines the direction of the recommendation is (surgical and operating room staff) expertise. External validity of relevant research evidence is determined by the degree of expertise of surgeons and operating room staff. Consensus reports detailing training and considerations on expertise can be found here [11].

See Table 1 and full content in MAGICapp.

**Table 1:** Evidence summary on Q1: TaTME versus laparoscopic TME

Outcome Timeframe	Study results and measurements	Absolute effect estimates		Certainty of the Evidence (Quality of evidence)	Plain language summary
		Lap TME	TaTME		
Major morbidity^a^ 30 days	Odds Ratio: 0.81 (CI 95% 0.47—1.39) Based on data from 550 patients in 7 studies^b^ Follow up 30 days	**120** per 1000	**99** per 1000	**Moderate** Due to serious risk of bias^c^	TaTME may have little or no effect on major morbidity
		Difference: **21 fewer per 1000** (CI 95% 60 fewer—39 more)			
Minor morbidity^d^ 30 days	Odds Ratio: 0.87 (CI 95% 0.52—1.44) Based on data from 486 patients in 6 studies^e^ Follow up 30 days	**160** per 1000	**142** per 1000	**Moderate** Due to serious risk of bias^f^	TaTME may have little or no effect on minor morbidity
		Difference: **18 fewer per 1000** (CI 95% 70 fewer—55 more)			
Mortality^g^ 30 days	Odds Ratio: 0.27 (CI 95% 0.08—0.88) Based on data from 1859 patients in 11 studies^h^ Follow up 30 days	**167** per 1000	**51** per 1000	**Low** Due to few events, due to serious risk of confounding bias^i^	TaTME may decrease 30-day or in-hospital mortality
		Difference: **116 fewer per 1000** (CI 95% 151 fewer—17 fewer)			
Anastomotic leakage^j^	Odds Ratio: 1.16 (CI 95% 0.82—1.63) Based on data from 1657 patients in 8 studies^k^ Follow up 30 days	**79** per 1000	**90** per 1000	**Low** Due to serious risk of bias and due to serious imprecision^l^	TaTME may have little or no effect on anastomotic leakage
		Difference: **11 more per 1000** (CI 95% 13 fewer—44 more)			
Stoma construction^m^	Odds Ratio: 1.21 (CI 95% 0.56—2.63) Based on data from 1407 patients in 7 studies^n^	**596** per 1000	**641** per 1000	**Very low** Due to serious inconsistency and due to very serious imprecision^o^	We are uncertain whether TaTME increases or decreases odds of stoma construction
		Difference: **45 more per 1000** (CI 95% 144 fewer—199 more)			
TME completeness^p^	Odds Ratio: 1.9 (CI 95% 0.81—4.44) Based on data from 1415 patients in 7 studies^q^	**724** per 1000	**833** per 1000	**Low** Due to serious risk of bias and due to serious imprecision/inconsistency^r^	TaTME may have little or no effect on TME completeness
		Difference: **109 more per 1000** (CI 95% 44 fewer—197 more)			
Clear CRM^s^	Odds Ratio: 1.36 (CI 95% 0.88—2.08) Based on data from 1909 patients in 12 studies^t^	**945** per 1000	**959** per 1000	**Moderate** Due to serious risk of bias^u^	TaTME probably has little or no effect on clear CRM
		Difference: **14 more per 1000** (CI 95% 7 fewer—28 more)			
Clear DRM^v^	Odds Ratio: 1.51 (CI 95% 0.7—3.24) Based on data from 1521 patients in 8 studies^w^	**981** per 1000	**987** per 1000	**Moderate** Due to serious risk of bias^x^	TaTME probably has little or no effect on clear DRM
		Difference: **6 more per 1000** (CI 95% 8 fewer—13 more)			
Low anterior resection syndrome	Odds Ratio: 0.63 (CI 95% 0.1—4.21) Based on data from 46 patients in 1 study^y^ Follow up 6 months	**913** per 1000	**869** per 1000	**Very low** Due to very serious imprecision and due to serious inconsistency^z^	We are uncertain whether TaTME increases or decreases odds of low anterior resection syndrome. There was inconsistency in reported effect by panel members
		Difference: **44 fewer per 1000** (CI 95% 401 fewer—65 more)			
Local recurrence 2 years	Hazard Ratio: 0.4 (CI 95% 0.23—0.69) Based on data from 710 patients in 1 study^aa^ Follow up 3 years	**96** per 1000	**40** per 1000	**Low** Due to serious risk of bias, due to serious imprecision^ab^	TaTME may decrease local recurrence
		Difference: **56 fewer per 1000** (CI 95% 73 fewer—29 fewer)			
Overall survival 5 years	Hazard Ratio: 0.74 (CI 95% 0.53—1.03) Based on data from 710 patients in 1 study^ac^ Follow up 3 years	**178** per 1000	**135** per 1000	**Very low** Due to serious risk of bias, due to serious indirectness, due to serious imprecision^ad^	We are uncertain whether TaTME increases or decreases overall survival
		Difference: **43 fewer per 1000** (CI 95% 79 fewer—5 more)			
Disease-free survival 5 years	Hazard Ratio: 0.81 (CI 95% 0.65—1.02) Based on data from 710 patients in 1 study^ae^ Follow up 3 years	**314** per 1000	**263** per 1000	**Very low** Due to serious risk of bias, due to serious indirectness, due to serious imprecision^af^	We are uncertain whether TaTME increases or decreases disease-free survival
		Difference: **51 fewer per 1000** (CI 95% 97 fewer—8 more)			
Quality of life	Based on data from 54 patients in 1 study^ag^ Follow up 6.6 months	Only one study at critical risk of bias reports on quality of life		**Very low** Due to very serious risk of bias and due to very serious imprecision^ah^	We are uncertain whether TaTME improves or worsens quality of life. There was inconsistency in reported effect by panel members

^1^30-day complications Clavien-Dindo ≥ 3

^2^Systematic review with included studies: [29, 30, 32, 36, 41–43] **Baseline/comparator** Control arm of reference used for intervention

^3^**Risk of Bias: no serious.** Due to serious risk of bias in measurement of outcome

^4^30-day complications Clavien-Dindo ≤ 2

^5^Primary study [29, 32, 36, 41–43] **Baseline/comparator** Control arm of reference used for intervention

^6^**Risk of Bias: no serious.** Due to serious risk of bias in outcome measurement

^7^30-day or in-hospital mortality

^8^Primary study [29–32, 34, 36–38, 41–43] **Baseline/comparator** Control arm of reference used for intervention

^9^**Risk of Bias: serious.** Due to confounding. **Imprecision: serious.** Due to few events

^10^Anastomotic leakage, as defined by the primary study authors, including pelvic abscess, purulent drain discharge, operative findings of anastomotic leakage, etc. This outcome is encompassed by the outcomes 'major morbidity' and 'minor morbidity', therefore it was not considered as an independent outcome in the evidence-to-decision framework.

^11^Primary study [29–31, 34, 37, 38, 41, 43] **Baseline/comparator** Control arm of reference used for intervention

^12^**Risk of Bias: no serious.** due to bias in outcome measurement. **Imprecision: serious.** Due to wide confidence intervals beyond panel-set minimal important difference.

^13^Patients with either protective ileostomy or Hartmann's procedure as cases with stoma

^14^Primary study [29–32, 36, 37, 43] **Baseline/comparator** Control arm of reference used for intervention

^15^**Risk of Bias: no serious.** due to confounding. **Inconsistency: serious.** Point estimates vary widely, the magnitude of statistical heterogeneity was high, with I^2: 83%, the direction of the effect is not consistent among the included studies. **Imprecision: very serious.** Due to wide confidence intervals beyond panel-set minimal important difference. We decided to not downgrade for both inconsistency and imprecision; however, we double-downgraded for very serious imprecision

^16^Completeness of TME assessed using the Quirke criteria.

^17^Primary study [29, 30, 32, 34, 37, 42, 44] **Baseline/comparator** Control arm of reference used for intervention

^18^**Risk of Bias: no serious.** Due to confounding. **Inconsistency: serious.** Point estimates vary widely, the confidence interval of some of the studies do not overlap with those of most included studies/ the point estimate of some of the included studies, the direction of the effect is not consistent among the included studies, the magnitude of statistical heterogeneity was high, with I^2: 84%. **Imprecision: serious.** Wide confidence intervals beyond panel-set minimal important difference. We decided to not downgrade for both inconsistency and imprecision. **Publication bias: no serious**

^19^Tumor-free circumferential resection margin at a distance of at least 1 mm

^20^Primary study [29–34, 36–38, 41–43] **Baseline/comparator** Control arm of reference used for intervention

^21^**Risk of Bias: no serious.** Due to confounding

^22^Tumor-free distal resection margin at a distance of at least 1 mm

^23^Primary study [29, 30, 32, 34, 37, 38, 42, 44] **Baseline/comparator** Control arm of reference used for intervention

^24^**Risk of Bias: serious.** Due to confounding

^25^Primary study [42] **Baseline/comparator** Control arm of reference used for intervention

^26^**Risk of Bias: no serious.** Due to confounding of the observational study. Expert-based evidence. **Inconsistency: serious.** Inconsistent opinion of panel members. **Indirectness: no serious.** Panel's input: Not substantial deviation from common practice, rather representative of variations. **Imprecision: very serious.** Wide confidence intervals, low number of patients, only data from one study. Possible recall bias by panel members

^27^Primary study [44] **Baseline/comparator** Control arm of reference used for intervention

^28^**Risk of Bias: serious.** Incomplete data and/or large loss to follow up. **Imprecision: serious.** Due to small number of events

^29^Primary study [44] **Baseline/comparator** Control arm of reference used for intervention

^30^**Risk of Bias: serious.** Incomplete data and/or large loss to follow up. **Indirectness: serious.** The outcome time frame in studies was insufficient. **Imprecision: serious.** Low number of patients, wide confidence intervals beyond panel-set minimal important difference

^31^Primary study [44] **Baseline/comparator** Control arm of reference used for intervention

^32^**Risk of Bias: serious.** Incomplete data and/or large loss to follow up. **Indirectness: serious.** The outcome time frame in studies was insufficient. **Imprecision: serious.** Low number of patients, wide confidence intervals beyond panel-set minimal important difference

^33^Primary study Supporting references [35]

^34^**Risk of Bias: very serious.** Incomplete data and/or large loss to follow up, due to risk of bias in outcome measurement. Expert-based evidence. **Indirectness: no serious.** Not substantial deviation from common practice, rather representative of variations. **Imprecision: very serious.** Wide confidence intervals, only data from one study. Possible recall bias by panel members

### Recommendation – TaTME versus robotic TME

**We suggest TaTME over robotic TME if expertise is available.** *Weak recommendation*

*Rationale*

The panel recognized that the evidence was very limited to allow assessment of the balance between benefits and harms with confidence. Several panel members suggested that surgeon's expertise plays a vital role and probably affects outcomes, so that both options may be appropriate. Substantial variability in patient values and preferences is anticipated and shared decision making after discussion of surgeon's preference and expertise, and perceived benefits and harms is encouraged. There is uncertainty around the use of resources, which depends on whether robotic-assisted or laparoscopic-assisted TaTME is performed, and on the selection between disposable or reusable instruments for laparoscopic-assisted TaTME. Equity might be reduced, due to lack of widespread expertise and longer use of operating room resources, at least during the early stages of implementation. The panel considered the intervention to be acceptable to key stakeholders, whereas feasibility was considered to vary and depend on annual volume of cases and centralization of care. Consensus reports detailing training and considerations on expertise can be found here [11].

See Table 2 and full content in MAGICapp.

**Table 2:** Evidence summary on Q2: TaTME versus robotic TME

Outcome Timeframe	Study results and measurements	Absolute effect estimates		Certainty of the Evidence (Quality of evidence)	Plain text summary
		Robotic TME	TaTME		
Mortality^a^ 30 days	Odds Ratio: 0.33 (CI 95% 0.02—6.81) Based on data from 596 patients in 1 study^b^ Follow up 30 days	**5** per 1000	**2** per 1000	**Very low** Due to very serious imprecision^c^	We are uncertain whether TaTME increases or decreases mortality
		Difference: **3 fewer per 1000** (CI 95% 5 fewer—28 more)			
Anastomotic leakage^d^	Odds Ratio: 1.12 (CI 95% 0.65—1.91) Based on data from 596 patients in 1 study^e^	**100** per 1000	**111** per 1000	**Very low** Due to serious risk of bias and due to very serious imprecision^f^	We are uncertain whether TaTME increases or decreases odds of anastomotic leakage
		Difference: **11 more per 1000** (CI 95% 33 fewer—75 more)			
Stoma construction^g^	Odds Ratio: 3.6 (CI 95% 1.97—6.55) Based on data from 596 patients in 1 study^h^	**808** per 1000	**938** per 1000	**Very low** Due to very serious imprecision^i^	We are uncertain whether TaTME increases or decreases odds of stoma construction
		Difference: **130 more per 1000** (CI 95% 84 more—157 more)			
TME completeness^j^	Odds Ratio: 0.48 (CI 95% 0.23—1.0) Based on data from 596 patients in 1 study^k^	**962** per 1000	**924** per 1000	**Very low** Due to very serious imprecision^l^	We are uncertain whether TaTME increases or decreases odds of TME completeness
		Difference: **38 fewer per 1000** (CI 95% 109 fewer—0 fewer)			
Clear CRM^m^	Odds Ratio: 1.07 (CI 95% 0.52—2.23) Based on data from 596 patients in 1 study^n^	**943** per 1000	**947** per 1000	**Very low** Due to very serious imprecision^o^	We are uncertain whether TaTME increases or decreases odds of clear CRM
		Difference: **4 more per 1000** (CI 95% 47 fewer—31 more)			
Clear DRM^p^	Odds Ratio: 0.15 (CI 95% 0.02—1.35) Based on data from 596 patients in 1 study^q^	**997** per 1000	**980** per 1000	**Very low** Due to very serious imprecision^r^	We are uncertain whether TaTME increases or decreases odds of clear DRM
		Difference: **17 fewer per 1000** (CI 95% 128 fewer—1 more)			
Major morbidity^s^ 30 days					No studies were found that looked at major morbidity
Minor morbidity^t^ 30 days					No studies were found that looked at minor morbidity
Local recurrence^u^ 2 years					No studies were found that looked at local recurrence at 2 years
Overall survival 5 years					No studies were found that looked at 5-year overall survival
Disease-free survival 5 years					No studies were found that looked at 5-year disease-free survival
Low anterior resection syndrome					No studies were found that looked at low anterior resection syndrome
Quality of life					No studies were found that looked at quality of life

^1^30-day or in-hospital mortality

^2^Primary study [41] **Baseline/comparator** Control arm of reference used for intervention

^3^**Imprecision: Very serious.** Wide confidence intervals, low number of patients, only data from one study

^4^Anastomotic leakage, as defined by the primary study authors, including pelvic abscess, purulent drain discharge, operative findings of anastomotic leakage, etc. This outcome is encompassed by the outcomes 'major morbidity' and 'minor morbidity'; therefore it was not considered as an independent outcome in the evidence-to-decision framework

^5^Primary study [41] **Baseline/comparator** Control arm of reference used for intervention

^6^**Risk of Bias: No serious.** Due to risk of bias in outcome measurement. **Imprecision: Very serious.** Wide confidence intervals, low number of patients, only data from one study

^7^Patients with either protective ileostomy or Hartmann's procedure as cases with stoma

^8^Primary study [41] **Baseline/comparator** Control arm of reference used for intervention

^9^**Imprecision: Very serious.** Only data from one study

^10^Completeness of TME assessed using the Quirke criteria

^11^Primary study [41] **Baseline/comparator** Control arm of reference used for intervention

^12^**Imprecision: Very serious.** Wide confidence intervals beyond panel-set minimal important differences, only data from one study

^13^Tumor-free circumferential resection margin at a distance of at least 1 mm

^14^Primary study [41] **Baseline/comparator** Control arm of reference used for intervention

^15^**Imprecision: Very serious.** Wide confidence intervals, only data from one study

^16^Tumor-free distal resection margin at a distance of at least 1 mm

^17^Primary study [41] **Baseline/comparator** Control arm of reference used for intervention

^18^**Imprecision: Very serious.** Wide confidence intervals, only data from one study

^19^30-day complications Clavien-Dindo ≥ 3

^20^30-day complications Clavien-Dindo ≤ 2

^21^30-day complications Clavien-Dindo ≤ 2

## Discussion

### Implications for policy makers

TaTME represents an option for the treatment of low rectal cancer, next to laparoscopic and robotic rectal resection. Although evidence on economic considerations is limited, empirical evidence does not suggest increased overall cost. Centralization of rectal cancer management may be necessary to allow accumulation of experience, which may play a vital role in operative outcomes.

### Implications for healthcare professionals

Surgeons with experience in TaTME are not advised against performing TaTME in patients with low rectal cancer, as evidence from comparative observational studies which have adjusted for confounders does not indicate increased harm, moreover there is evidence of moderate certainty suggesting lower 30-day mortality and lower rate of recurrence at 3 years.

Substantial new evidence is awaited within the next few years, so that surgeons who are not trained in TaTME may not change their practice for the present. Importantly, evidence considered in this rapid guideline derives primarily from centers and surgeons with experience in TaTME; guideline users are therefore advised to exercise caution in extrapolating the evidence summarized herein.

### Implications for patients

Patients can be informed that available evidence suggests similar outcomes between TaTME and laparoscopic TME, whereas 30-day mortality and 3-year loco-regional recurrence may be lower with TaTME if the surgeon has experience with this technique. Furthermore, they may want to discuss expected benefits and potential harms, and their surgeon's experience and preference.

### Implications for researchers

There are important gaps in evidence, which are expected to be addressed by future research:

*TaTME v. laparoscopic TME* De novo RCTs may not be necessary, because several trials are currently underway and their results are expected to be published within the next years (see *Validity period* below). Matched cohort studies are needed to address the outcomes major morbidity, 30-day or in-hospital mortality, 2-year recurrence, 5-year disease-free and overall survival, low anterior resection syndrome and quality of life. Importantly, further reports of unmatched cohorts do not contribute reliable information to the body of evidence and may be redundant and potentially misleading. Researchers may want to consider performing analyses that have adjusted for sex, BMI, ASA classification, tumor stage and distance from anal verge, and neoadjuvant chemoradiotherapy. To reach sufficient sample size, multi-institutional collaborations or registry analyses are encouraged. Analyses of male patients, patients who underwent neoadjuvant chemoradiotherapy and level (height) up to which transanal dissection was performed are expected to address the outcomes of TaTME in these subgroups.

*TaTME v. robotic TME* Available evidence is extremely limited and the same research considerations apply here as well. Critical and important outcomes as listed in the *Methods* section are expected to be addressed.

### Monitoring

Use of the guideline by EAES members will be monitored through an online survey 2 years after publication. Feedback from target users in the form of email communication, letters to the editor, and comments in social media will be documented to be addressed by future versions.

### Validity period

A scoping search of ClinicalTrials.gov, EU Clinical Trials Register, WHO International Clinical Trials Registry Platform, EORTC and ISRCTN registry identified at least 5 ongoing RCTs comparing TaTME with laparoscopic (*n* = 4) or robotic (*n* = 1) TME, including two mega-trials (planned to recruit > 1000 patients each) [54–58]. Completion dates range from June 2021 to July 2025. Under consideration of the reported follow-up duration of critical outcomes, substantial new evidence is expected by 2025 for Q1 and by 2026 for Q2. The validity of the present version of this rapid guideline is set until December 2025. Please read the *Disclaimer* for further information regarding validity.

### Update

An update of this rapid guideline is planned to take place in 2025. However, one could anticipate a change in the direction or the strength of the recommendation when data from cohort studies or registries become available, under the condition that their methodological quality will be high. The EAES Research Committee/Guidelines Subcommittee will keep monitoring new evidence and update this document if such data become published.

## Conclusion

This rapid review summarizes highest quality evidence and provides evidence-based and trustworthy recommendations on the use of TaTME for low rectal cancer.

## Supplementary Information

Below is the link to the electronic supplementary material.

Supplementary file1 (DOCX 1980 KB)

## Declarations

### Disclaimer

This clinical practice guideline has been developed under the auspice of the European Association for Endoscopic Surgery (EAES). It is intended to be used primarily by health professionals (e.g., surgeons, anesthetists, physicians) and to assist in making informed clinical decisions on diagnostic measures and therapeutic management. It is also intended to inform individual practice of allied health professionals (e.g., surgical nurses, dieticians, physical rehabilitation therapists, psychologists); to inform strategic planning and resource management by health care authorities (e.g., regional and national authorities, health care institutions, hospital administration authorities); and to inform patients wishing to obtain an overview of the condition of interest and its management.

The use of recommendations contained herein must be informed by supporting evidence accompanying each recommendation and by research evidence that might not have been published by the time of writing the present document. Users must, thus, base their actions informed by newly published evidence at any given point in time.

The information in the guideline should not be relied upon as being complete or accurate, nor should it be considered as inclusive of all proper treatments or methods of care or as a statement of the standard of care. With the rapid development of scientific knowledge, new evidence may emerge between the time the guideline is developed and when it is published or read. The guideline is not continually updated and may not reflect the most recent evidence. The guideline addresses only the topics specifically identified therein and is not applicable to other interventions, diseases, or stages of diseases. This guideline does not mandate any particular course of medical care. Further, the guideline is not intended to substitute the independent professional judgment of the treating provider, as the guideline does not necessarily account for individual variation among patients.

Even if evidence on a topic suggests a specific diagnostic and/or treatment action, users and especially health professionals may need to decide against the suggested or recommended action in view of circumstances related to patient values, preferences, co-morbidities and disease characteristics; available human, monetary and material resources; and healthcare infrastructures.

EAES provides this guideline on an “as is” basis, and makes no warranty, express or implied, regarding the guideline.
