# Supplementary material for: UEG and EAES rapid guideline: Systematic review, meta-analysis, GRADE assessment and evidence-informed European recommendations on TaTME for rectal cancer
Source: Surg Endosc. 2022 Feb 25;36(4):2221–32. doi: 10.1007/s00464-022-09090-4 (PMC8921163; doi:10.1007/s00464-022-09090-4)

**APPENDIX**

**to**

**EAES Rapid Guideline: TaTME for Rectal Cancer**

**COMMENTS TO THE LITERATURE SEARCH PROCESS**

First-level and second level screening were performed by two reviewers independently (SAA, MM) using the platform Rayyan. Both reviewers were blinded to the other reviewer's judgement. Conflicts were encountered in 0.9% of the records (n=7), which were resolved by discussion.

For a substantial proportion of records we could not identify the full texts. This was typically because they referred to abstracts of congress presentations, rather than lack of access to the full text. We did not consider data provided in abstract form, because these do not provide sufficient information to allow for risk of bias assessment, hence they would be excluded from further analysis as per ROBINS-I (critical risk of bias due to insufficient information) [1]. Detailed reasons for exclusion can be found online [2].

Particular care was taken to avoid inclusion of studies reporting on overlapping patient populations, by cross checking the country and institution, the authors' names, any registries where patient data were provided to, years of patient recruitment, etc. Pathological outcomes (TME completeness, clear DRM) of a number of reports were excluded [3-7] , due to overlapping patient populations with a multicenter study, as indicated by the primary author of the latter [44] . Outcomes from these original studies that were not reported cumulatively by the multicenter study were properly included in the analyses. For the outcome clear CRM, we included the original studies and not the multicenter study, because the cumulative number of patients was smaller in the multicenter study. More detailed information is provided in the data extraction sheet in the online appendix [2].

**AMENDMENTS TO THE PROTOCOL**

First and second level screening was carried out by two investigators, instead of one and cross-checking by another investigator, to increase the sensitivity of the search.

Interrogation of medical databases identified cohort studies only. ROBINS-I suggests that studies at critical risk of bias should not be included in evidence synthesis [1]. We considered studies that did not adjust for important confounders to be at critical risk of bias due to confounding (hence, at critical overall risk of bias), and we did not consider them further for qualitative and quantitative analysis. These confounders were:

1. Sex
2. BMI
3. Tumor stage
4. Neoadjuvant CRT

This approach has the drawback that we cannot assess the possibility of substantial effect in spite of residual bias. Nevertheless, in our experience this is an extremely rare scenario and we have never encountered this in the surgical literature. Furthermore, evidence from studies adjusting for the above confounders (in the TaTME v. lap. TME comparison) does not suggest that effect despite residual confounding would be a plausible scenario in this context.

Following feedback from EAES members, we planned to perform the following subgroup analyses (in addition to the pre-specified ones), however no relevant data were available:

1. Male patients
2. Patients with neoadjuvant radiotherapy
3. Level of transanal dissection

**COMMENTS TO DATA EXTRACTION AND OUTCOME ASSESSMENT**

Outcome data were extracted from one author (MM) and cross checked by a second author (SAA). Disagreements were resolved by discussion. Risk of bias assessments were performed by one author (SAA).

Stoma construction was considered both as co-intervention and as confounder in risk of bias assessment, in studies where stoma construction rates differed substantially between the intervention and the comparator groups.

We considered patients with either protective ileostomy or Hartmann's procedure as cases with stoma, whenever this information was provided.

Completeness of TME was assessed using the Quirke criteria across studies [9]. There was no substantial variability among studies with regard to circumferential and distal resection margin assessment; the authors typically considered a distance of >1mm between the tumor (or the deepest tumor invasion) and the circumferential or distal resection margin to indicate no margin involvement.

We added the outcome 2-year local recurrence based on panel's and EAES members' input. The importance of this outcome was determined during the consensus meeting. The minimal importance difference was decided upon after panel's online anonymous voting.

Risk of bias summary tables and graphs were constructed using the *robvis* tool [10].

**COMMENTS TO STATISTICAL ANALYSES**

We conducted a random effect meta-analysis to synthesize quantitatively the evidence for the two guideline questions since we expected much variation in the PICO criteria across trials [11]. All the included outcomes are dichotomous. From each study, we extracted the number of events and the sample size of each group, and we estimated for each outcome the odds ratio along with the corresponding 95% confidence interval. We used the method of moments estimator, also known as the DerSimonian & Laird estimator for the between study-variance (heterogeneity) [12]. A continuity correction was applied to the studies with zero-cell counts. We explored heterogeneity via the I^2^ statistic that describes the percentage of the variability of effect estimates, that is due to heterogeneity rather than sampling error. We further explored heterogeneity by computing the Q-statistic and the 95% predictive intervals that show the plausible range of effect size values for a future trial. It has been suggested that at least ten studies are needed for the Eggers’ test to be applied [13]. It was not possible to check for small study effect either visually by inspecting the symmetry of the funnel plot or statistically by applying the Egger’s test because of an inadequate number of studies for the majority of comparisons. The fixed effect (also known as common effect) model was applied for all the analyses as a sensitivity analysis. Yet, there were not important differences observed between the results of fixed and the random effects model. All the analyses were performed in R statistical package version 4.0.3 using the meta, metafor packages.

**COMMENTS TO ASSESSMENT OF THE CERTAINTY OF EVIDENCE**

For time-to-event outcomes, we planned to use the GRADE guidance on rating the certainty in time-to-event outcomes [14]. One study only provided data on loco-regional recurrence and survival outcomes, with a follow-up of 3 years [8]. The predefined outcomes were considered critical at 5-year follow-up, we therefore downgraded for indirectness. Further considerations are provided in the footnotes of the evidence tables.

**COMMENTS TO THE EVIDENCE-TO-DECISION FRAMEWORK**

Panel members were made aware that some outcomes are overlapping. Anastomotic leak is overlapping with Clavien-Dindo grouped outcomes. Negative circumferential margins and distal margins are contained within the outcome TME completeness. These 'subgroup' outcomes were not considered in the evidence-to-decision framework, relevant data are however provided because we considered they they would be valued by guideline users.

**REFERENCES**

1. Sterne JA, Hernán MA, Reeves BC, Savović J, Berkman ND, Viswanathan M, Henry D, Altman DG, Ansari MT, Boutron I, Carpenter JR, Chan AW, Churchill R, Deeks JJ, Hróbjartsson A, Kirkham J, Jüni P, Loke YK, Pigott TD, Ramsay CR, Regidor D, Rothstein HR, Sandhu L, Santaguida PL, Schünemann HJ, Shea B, Shrier I, Tugwell P, Turner L, Valentine JC, Waddington H, Waters E, Wells GA, Whiting PF, Higgins JP. ROBINS-I: a tool for assessing risk of bias in non-randomised studies of interventions. BMJ. 2016 Oct 12;355:i4919. doi: 10.1136/bmj.i4919. PMID: 27733354; PMCID: PMC5062054.
2. Antoniou SA. Appendix files for EAES Rapid Guideline: TaTME. 2021. Available in: <http://osf.io/65vkq>. Accessed July 15, 2021
3. Detering R, Roodbeen SX, van Oostendorp SE, Dekker J-WT, Sietses C, Bemelman WA, et al. Three-Year Nationwide Experience with Transanal Total Mesorectal Excision for Rectal Cancer in the Netherlands: A Propensity Score-Matched Comparison with Conventional Laparoscopic Total Mesorectal Excision. Journal of the American College of Surgeons 2019;228(3):235-244.e1
4. Velthuis S, Nieuwenhuis DH, Ruijter TEG, Cuesta MA, Bonjer HJ, Sietses C. Transanal versus traditional laparoscopic total mesorectal excision for rectal carcinoma. Surgical Endoscopy 2014;28(12):3494-9
5. Veltcamp Helbach M, Koedam TWA, Knol JJ, Velthuis S, Bonjer HJ, Tuynman JB, et al. Quality of life after rectal cancer surgery: differences between laparoscopic and transanal total mesorectal excision. Surgical Endoscopy 2019;33(1):79-87
6. Roodbeen SX, Penna M, Mackenzie H, Kusters M, Slater A, Jones OM, et al. Transanal total mesorectal excision (TaTME) versus laparoscopic TME for MRI-defined low rectal cancer: a propensity score-matched analysis of oncological outcomes. Surgical Endoscopy 2019;33(8):2459-2467
7. Fernández-Hevia M, Delgado S, Castells A, Tasende M, Momblan D, Díaz del Gobbo G, et al. Transanal total mesorectal excision in rectal cancer: short-term outcomes in comparison with laparoscopic surgery. Annals of Surgery 2015;261(2):221-7
8. de Lacy FB, Roodbeen SX, Ríos J, van Laarhoven J, Otero-Piñeiro A, Bravo R, Visser T, van Poppel R, Valverde S, Hompes R, Sietses C, Castells A, Bemelman WA, Tanis PJ, Lacy AM. Three-year outcome after transanal versus laparoscopic total mesorectal excision in locally advanced rectal cancer: a multicenter comparative analysis. BMC Cancer. 2020 Jul 20;20(1):677. doi: 10.1186/s12885-020-07171-y. PMID: 32689968; PMCID: PMC7372845
9. Quirke P, Durdey P, Dixon MF, Williams NS. Local recurrence of rectal adenocarcinoma due to inadequate surgical resection. Histopathological study of lateral tumour spread and surgical excision. Lancet (London, England) 1986;2(8514):996-9
10. McGuinness LA, Higgins JPT. Risk-of-bias VISualization (robvis): An R package and Shiny web app for visualizing risk-of-bias assessments. Research synthesis methods 2021;12(1):55-61
11. Nikolakopoulou A, Mavridis D, Salanti G. Demystifying fixed and random effects meta-analysis. Evid Based Ment Health. 2014 May;17(2):53-7. doi: 10.1136/eb-2014-101795. Epub 2014 Apr 1. Erratum in: Evid Based Ment Health. 2014 Aug;17(3):89. PMID: 24692250.
12. DerSimonian R, Laird N. Meta-analysis in clinical trials. Controlled Clinical Trials 1986;7(3):177-88
13. Egger M, Davey Smith G, Schneider M, Minder C. Bias in meta-analysis detected by a simple, graphical test. BMJ (Clinical research ed.) 1997;315(7109):629-34
14. Goldkuhle M, Bender R, Akl EA, van Dalen EC, Nevitt S, Mustafa RA, et al. GRADE Guidelines: 29. Rating the certainty in time-to- event outcomes-Study limitations due to censoring of participants with missing data in intervention studies. Journal of Clinical Epidemiology 2021;129 126-137

**APPENDIX FIGURES**

**PRISMA 2020 flowchart**


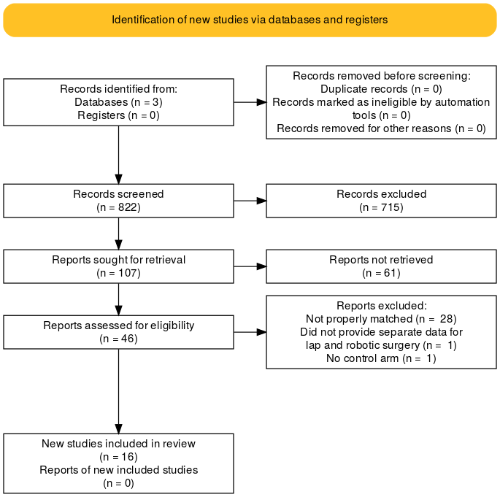


**Q1 Risk of bias summary 30-day complications Clavien-Dindo ≥3 (cohort studies)**


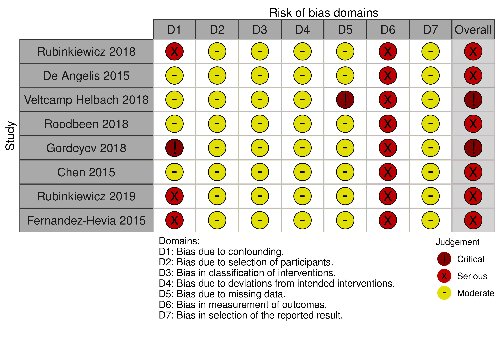


**Q1 Risk of bias graph 30-day complications Clavien-Dindo ≥3 (cohort studies)**


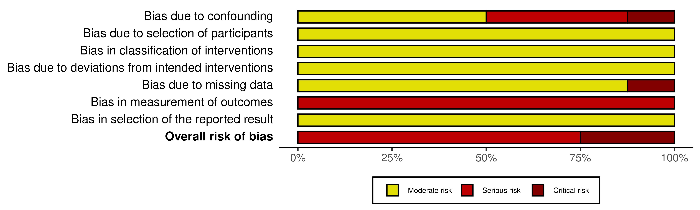


**Q1 Risk of bias summary 30-day complications Clavien-Dindo ≥3 (RCT)**


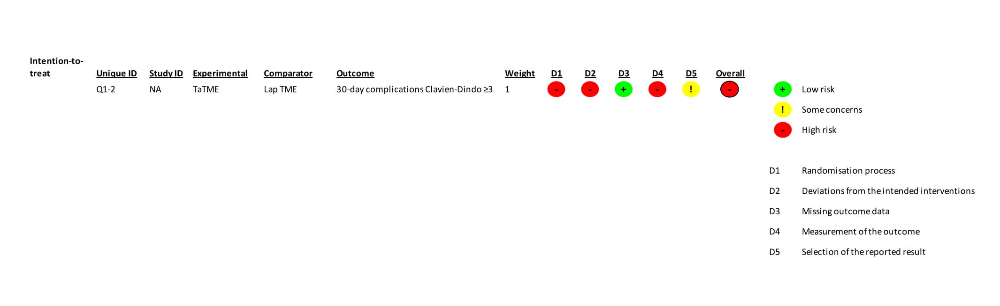


**Q1 Risk of bias summary 30-day complications Clavien-Dindo ≤2 (cohort studies)**


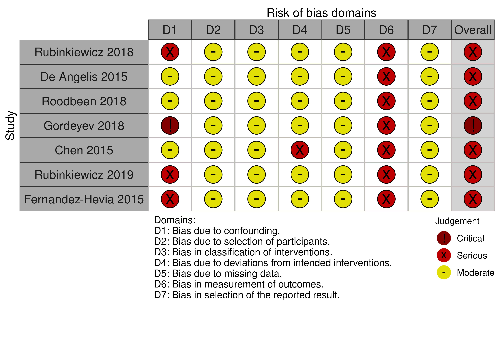


**Q1 Risk of bias graph 30-day complications Clavien-Dindo ≤2 (cohort studies)**


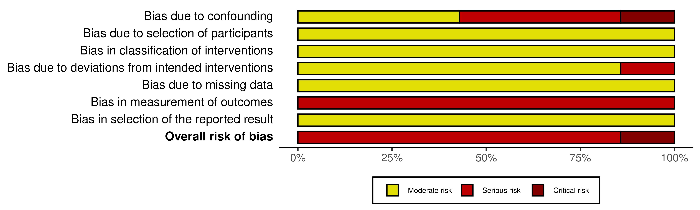


**Q1 Risk of bias summary 30-day mortality (cohort studies)**


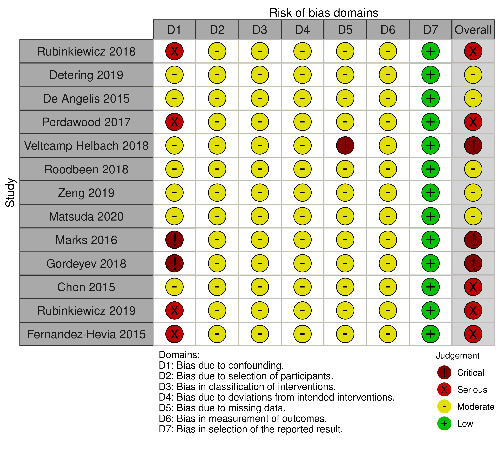


**Q1 Risk of bias graph 30-day mortality (cohort studies)**


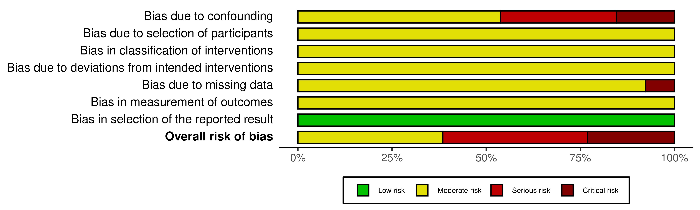


**Q1 Risk of bias summary 30-day mortality (RCT)**


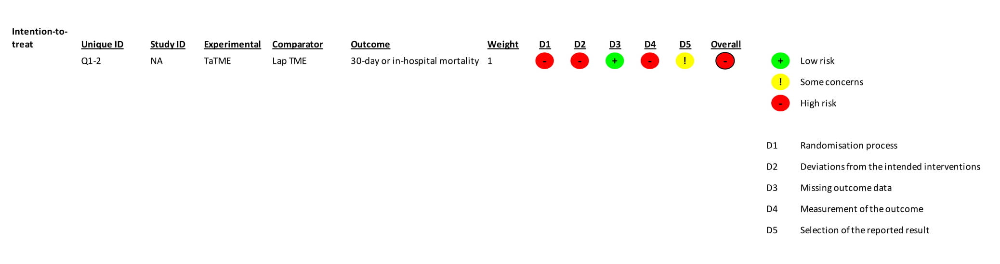


**Q2 Risk of bias summary 30-day mortality (cohort study)**


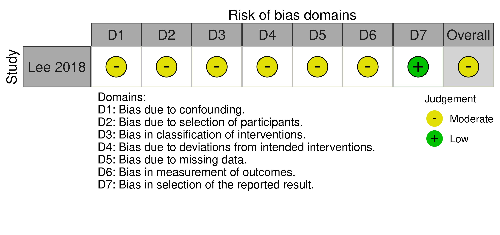


**Q1 Risk of bias summary anastomotic leakage (cohort studies)**


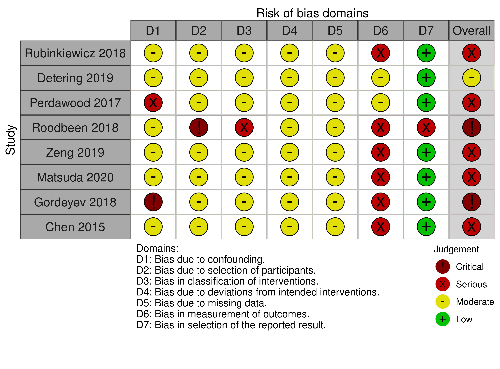


**Q1 Risk of bias graph anastomotic leakage (cohort studies)**


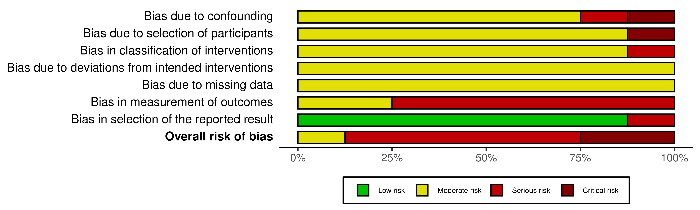


**Q1 Risk of bias summary anastomotic leakage (RCT)**


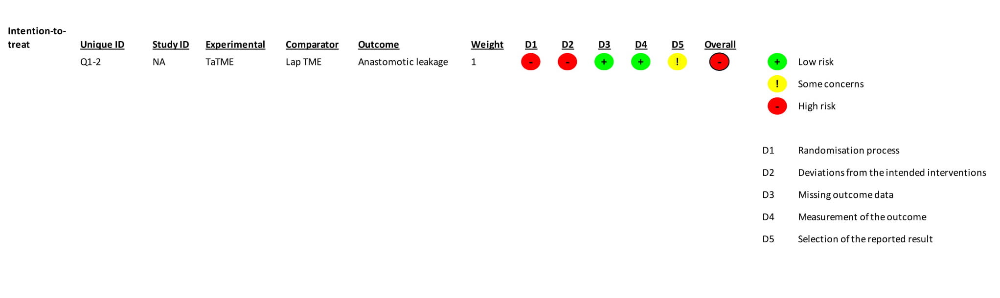


**Q2 Risk of bias graph anastomotic leakage (cohort study)**


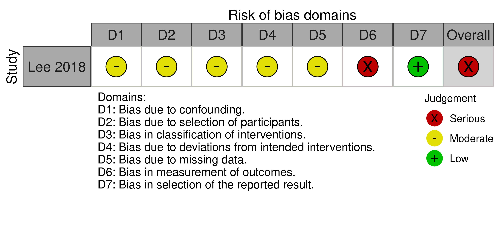


**Q1 Risk of bias summary stoma construction (cohort studies)**


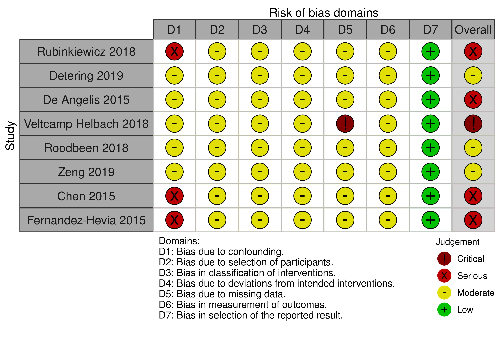


**Q1 Risk of bias graph stoma construction (cohort studies)**


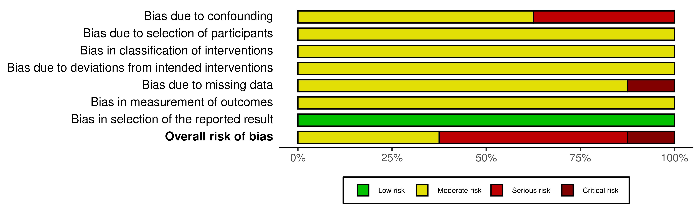


**Q2 Risk of bias summary stoma construction (cohort study)**


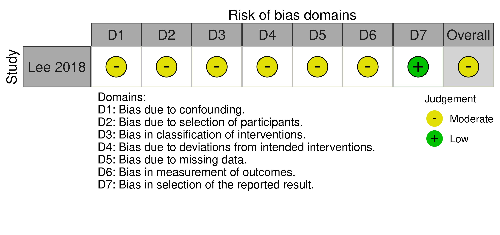


**Q1 Risk of bias summary pathological outcomes (cohort studies)**


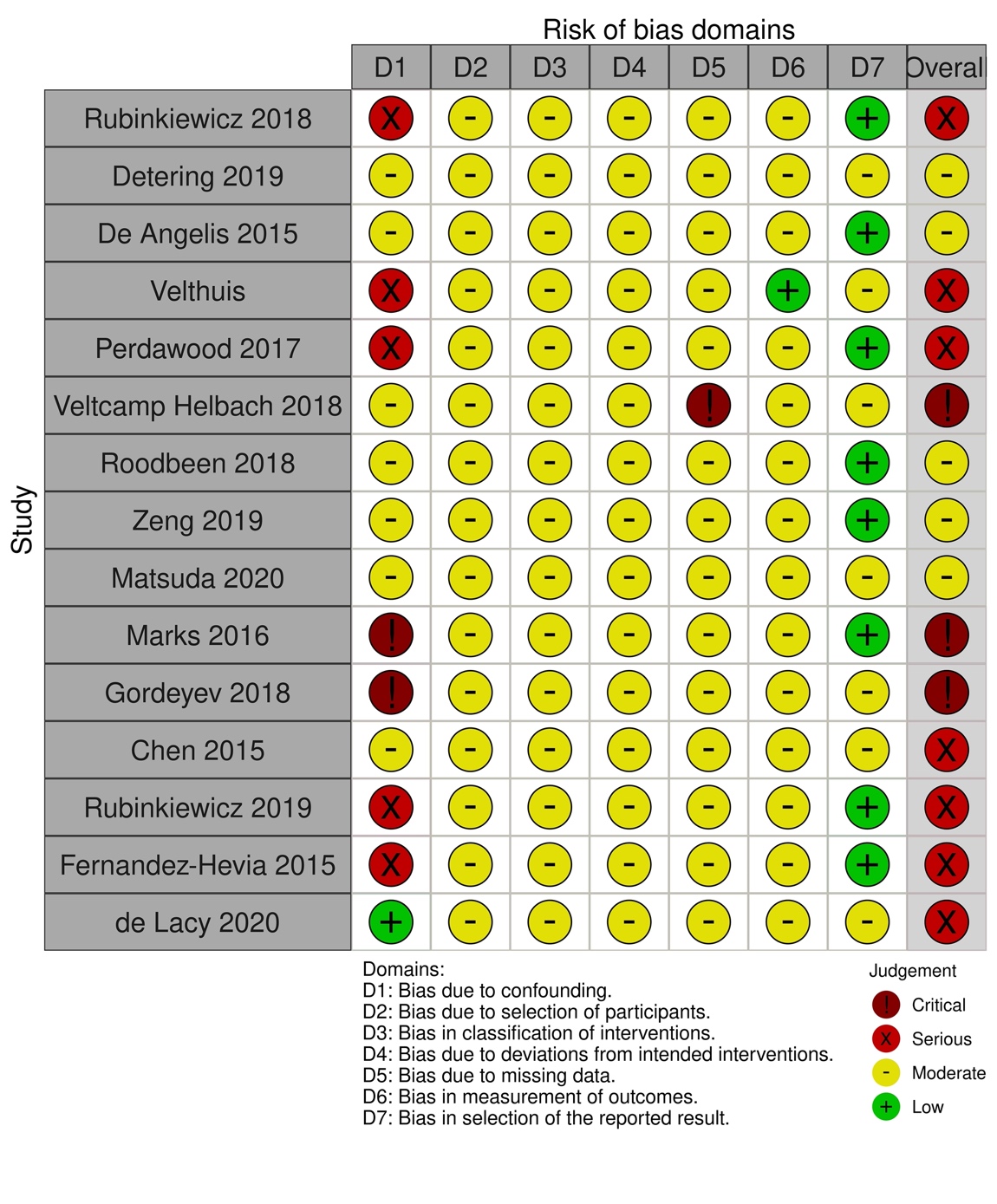


**Q1 Risk of bias graph pathological outcomes (cohort studies)**

 
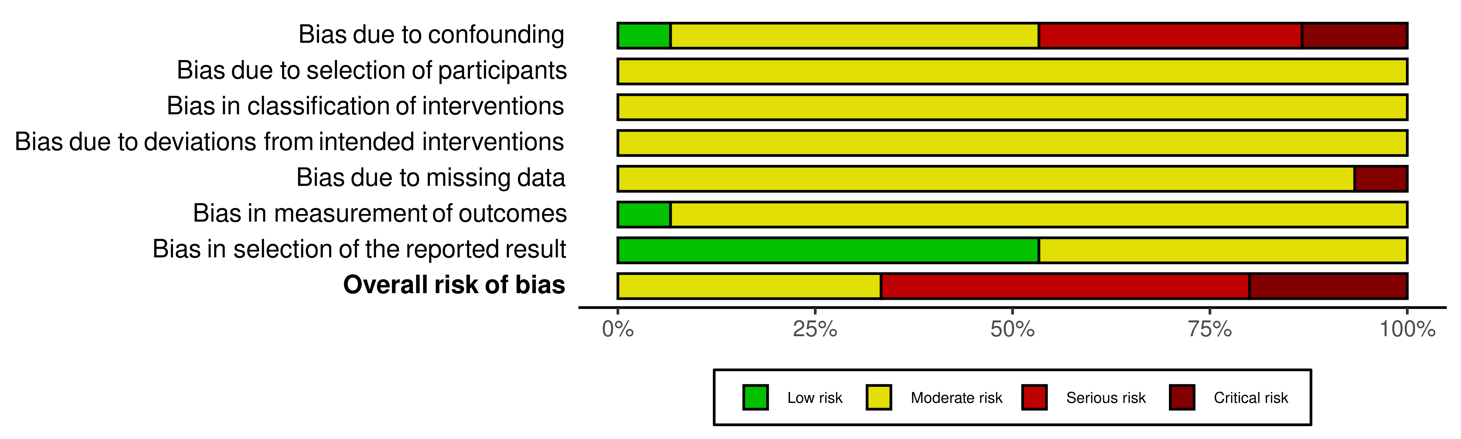


**Q2 Risk of bias summary pathological outcomes (cohort study)**


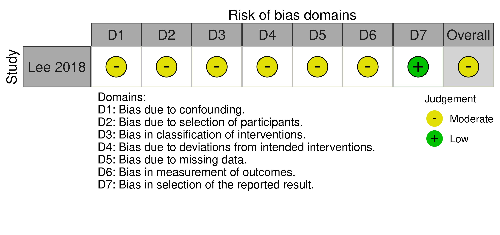


**Q1 Risk of bias summary local recurrence at 2 years (cohort studies)**

 
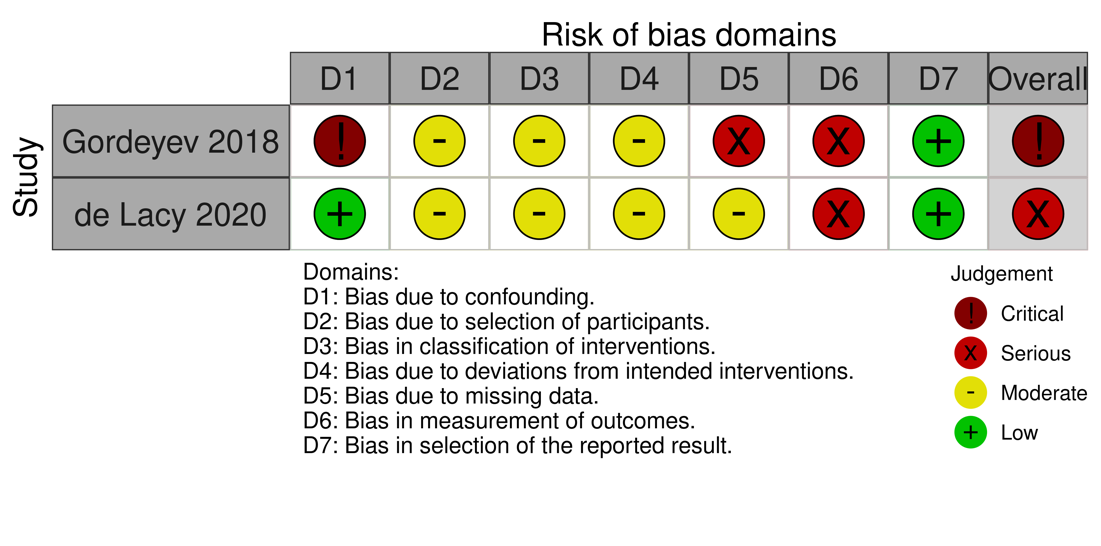


**Q1 Risk of bias graph local recurrence at 2 years (cohort studies)**


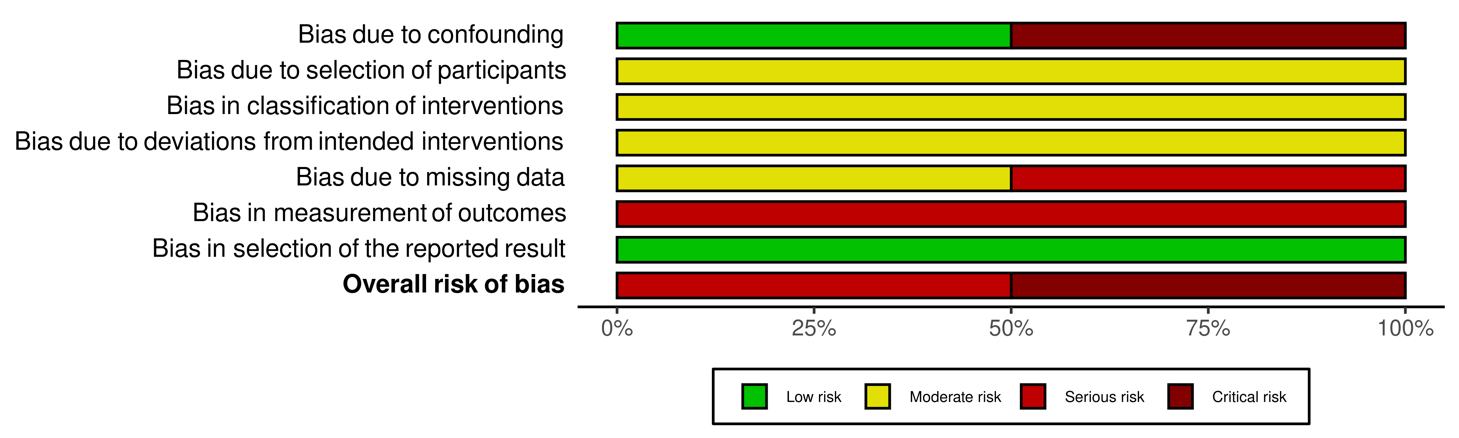


**Q1 Risk of bias summary 5-year overall survival (cohort studies)**


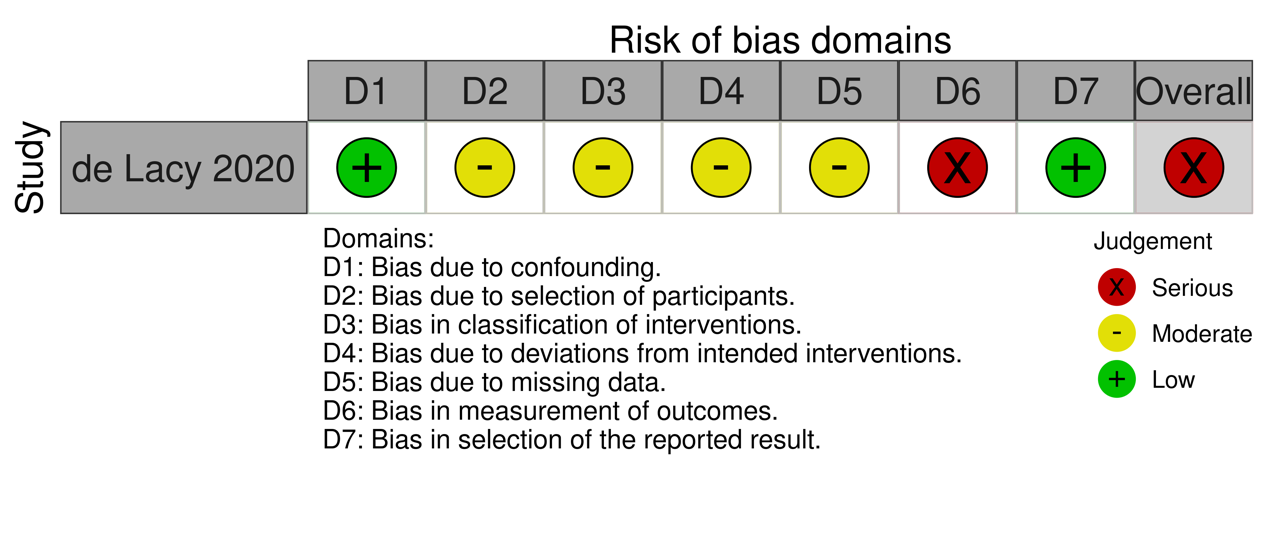


**Q1 Risk of bias summary 5-year disease-free survival (cohort studies)**


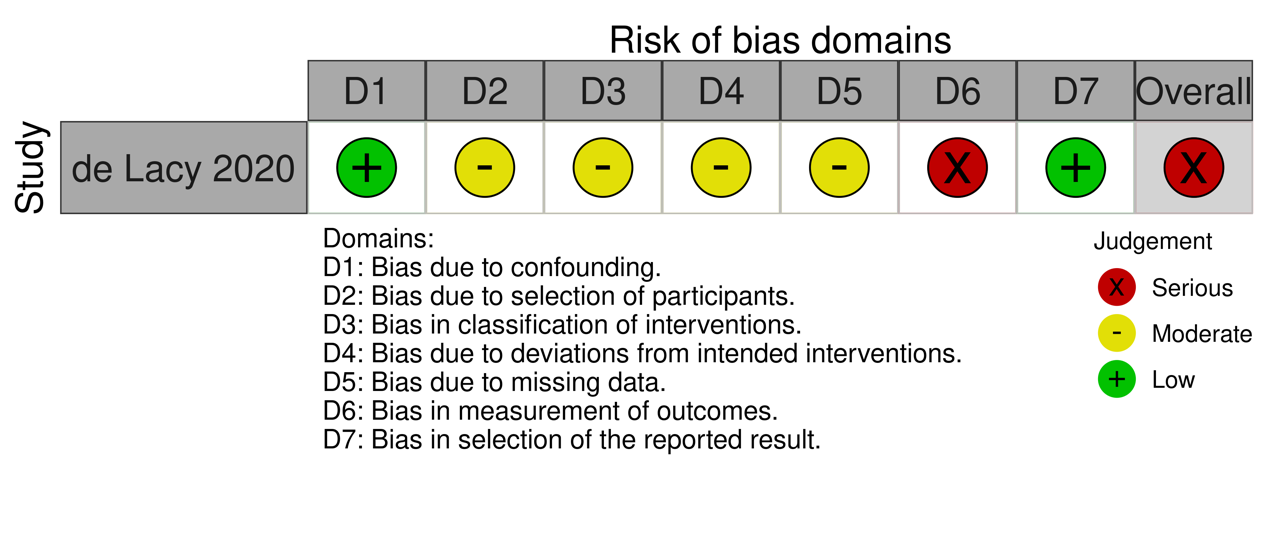


**Q1 Risk of bias summary low anterior resection syndrome (cohort studies)**


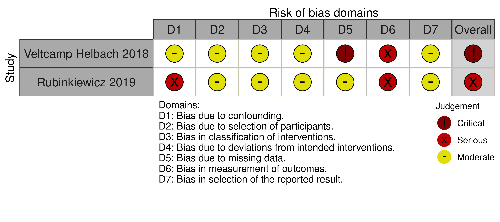


**Q1 Risk of bias graph low anterior resection syndrome (cohort studies)**


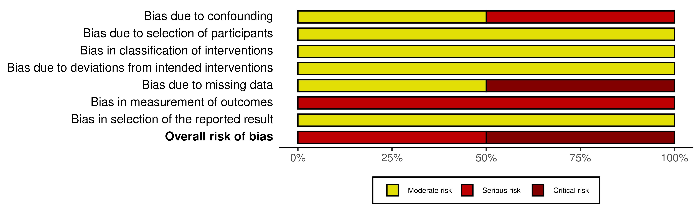


**Q1 Risk of bias summary quality of life (cohort study)**


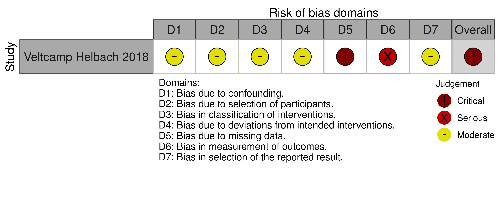

Supplement: Supplementary file 1 — Supplementary file1 (DOCX 1980 KB) [file 464_2022_9090_MOESM1_ESM.docx]
